# Extracellular Polymeric Substance-Intercalated MXene Membranes Toward Removal of Emerging Contaminants

**DOI:** 10.3390/membranes16060200

**Published:** 2026-06-08

**Authors:** Da-Qi Cao, Wen-Yu Qu, Yi-Xuan Song, Bi-Xiao Xu, Wen-Yu Zhang, Rongling Wu

**Affiliations:** 1 Sino-Dutch R&D Centre for Future Wastewater Treatment Technologies/Key Laboratory of Urban Stormwater System and Water Environment, Beijing University of Civil Engineering and Architecture, Beijing 100044, China; 2 Beijing Municipal Research Institute of Eco-Environmental Protection, Beijing 100037, China; 3 Shanghai Institute for Mathematics and Interdisciplinary Sciences, Shanghai 200433, China; 4 Beijing Key Laboratory of Topological Statistics and Applications for Complex Systems, Beijing Institute of Mathematical Sciences and Applications, Beijing 101408, China

**Keywords:** resource recovery, extracellular polymeric substance, MXene, emerging contaminant, membrane separation

## Abstract

Resource recovery from excess sludge, specifically the extraction of extracellular polymeric substances (EPSs), has become a frontier issue; yet achieving high-value utilization of this recovered resource remains a key bottleneck. Two-dimensional MXene membranes show great potential for emerging contaminants (ECs) separation owing to their lamellar structure and tunable surface chemistry. In this study, biological macromolecule (BM)-intercalated MXene (BM-M) composite membranes were fabricated using practical EPSs and model EPSs such as sodium alginate (SA), bovine serum albumin (BSA), and silk fibroin (SF) as sustainable intercalators. The interlayer spacing, surface charge, hydrophilicity, mechanical strength, functional group of BM-M membranes and their EC removal behaviors were systematically investigated. The practical EPS performed better than the model EPS, highlighting the importance of molecular complexity in interlayer design. The practical EPS-intercalated MXene (EPS-M) membrane achieved the removal efficiencies of 64.0%, 90.2% and 67.5% for diethyl phthalate (DEP), erythromycin (ERY) and sulfamethoxazole (SMX), respectively. The separation mechanism of ECs mainly included electrostatic, sieving, hydrophobic, and hydrogen bonding. This work highlights the effectiveness of EPS intercalation in tailoring MXene membrane structure for the removal of diverse ECs.

## 1. Introduction

The recovery and utilization of extracellular polymeric substances (EPSs) from excess sludge in wastewater treatment plants (WWTPs) has emerged as cutting-edge research [1,2,3,4]. EPS, as a natural biopolymer composite, has unique physicochemical properties such as adsorption, multifunctional groups, and tunable spatial structure [5,6,7]. Expanding the high value-added applications of EPSs has become a research hotspot [3,4]. From a resource recovery perspective, prioritizing EPS valorization over its conventional removal as waste offers added value to wastewater treatment.

Emerging contaminants (ECs), including persistent organic pollutants, microplastics, antibiotics, and endocrine disruptors, pose significant threats to ecosystems and human health due to their persistence, bioaccumulation potential, and inadequate removal by conventional wastewater treatment processes [8,9,10]. WWTPs are major discharge sources for these pollutants; however, established methods often prove insufficient for their effective elimination [11,12]. Their continued release and presence in aquatic environments underscore the urgent need for advanced remediation technologies [13,14,15,16].

Current strategies for EC removal encompass chemical, biological, and physical methods [17]. While chemical techniques like advanced oxidation and adsorption are the most effective techniques to target soluble pollutants, they may generate transformation products or cause secondary pollution [18]. Biological processes often struggle with the recalcitrance and potential toxicity of trace-level antibiotics and persistent organic compounds [14,19,20,21]. Conventional physical methods (e.g., screening, sedimentation) are primarily effective for suspended solids but inefficient against dissolved contaminants [17,22,23,24]. Membrane separation has emerged as a promising alternative, offering high removal efficiency and operational simplicity with relatively low energy consumption, though membrane material remains a notable limitation [9,10,12,25,26,27].

The performance of membrane technology is intrinsically linked to the development of advanced membrane materials [28]. Two-dimensional (2D) MXene membranes, characterized by their layered structure, high surface area, and tunable surface chemistry, demonstrate exceptional potential for separating ECs [29,30,31]. Intercalation with various agents (e.g., metal ions, aromatic compounds) can further enhance their separation efficiency, hydrophilicity, antifouling properties, and mechanical stability [3,32,33,34,35]. As noted by Alyasi et al. [36], while MXene membranes offer advantages like high throughput and rejection for specific ECs, challenges such as membrane fouling and limited efficiency for certain contaminants persist. Furthermore, EPS with their renewability, biocompatibility, and multifunctional groups, presents a sustainable strategy to enhance membrane hydrophilicity, stability, antifouling propensity, and selectivity [3].

Recent studies further underscore this potential. For instance, erythromycin (ERY) can be rejected with a removal rate of 92.9% using MXene membranes intercalated with columnar aromatic compounds [37]; Ti_3_C_2_T_X_ membranes on polyethersulfone (PES) substrates via vacuum-assisted filtration achieved an erythromycin removal rate of 95.0% [38]; tetracycline and meropenem were removed using PVDF/TiO_2_@MXene membranes, with removal rates of 87.8% and 60.7%, respectively [39]; tetracycline and sulfamethoxazole (SMX) were rejected using a MXene hybrid membrane via non-solvent induced phase separation, with removal rates of 97.2% and 42.0%, respectively [40]; per- and polyfluoroalkyl substances were eliminated with a removal rate of 95.0% using a mix-dimensional composite membrane embedding one-dimensional carboxylated cellulose nanofibers into a MXene lamellar structure [41]. Furthermore, Ti_3_C_2_T_X_-cellulose acetate membranes exhibited enhanced hydrophilicity and water flux along with effective organic matter removal [42].

Herein, practical EPS and three model biological macromolecules (BMs)—sodium alginate (SA, a polysaccharide), bovine serum albumin (BSA, a globular protein), and silk fibroin (SF, a fibrous protein)—were selected as intercalating agents to prepare BM-intercalated MXene (BM-M) membranes. These model BMs were chosen for their distinct molecular architectures and functionalities, enabling a structured comparison with the chemically complex practical EPS. Their performance was evaluated for the removal of three typical ECs such as diethyl phthalate (DEP), ERY, and SMX. These contaminants were selected to probe different removal mechanisms related to molecular size, hydrophobicity, and surface charge. The BM-M membranes were formed via constant-pressure dead-end filtration, and their properties, including interlayer spacing, surface charge, hydrophilicity, mechanical strength, and functional group were systematically characterized to correlate with contaminant removal efficiency.

## 2. Materials and Methods

### 2.1. Materials

The MAX phase (Ti_3_AlC_2_, 200 mesh) was purchased from Xinxi Technology Co., Ltd. (Foshan, China). Lithium fluoride (LiF) was supplied by Merck Group (Rahway, NJ, USA). Hydrochloric acid (HCl, 36–38%) was obtained from China National Pharmaceutical Group Co., Ltd. Polyvinylidene fluoride (PVDF) microfiltration membranes (0.22 μm) were provided by Longjing Membrane Technology Co., Ltd. (Jiangsu, China). Ultra-pure water (resistivity ≥ 18.2 MΩ·cm) was produced using an Arium Comfort II system (Sartorius, Germany). The BMs—SA (120–190 kDa), BSA (63 kDa), and SF (6–10 kDa)—were sourced from Sigma-Aldrich (USA). Target contaminants included DEP (≥99.5%, National Pharmaceutical Group, Beijing, China), ERY (≥98%, Macklin Inc., Shanghai, China), and SMX (≥98%, Chemical Industry Co., Ltd., Tokyo, Japan); their relevant properties are presented in detail in Table 1. Excess sludge was collected from a laboratory-scale University of Cape Town (UCT) reactor.

### 2.2. EPS Extraction

EPS was extracted from the sludge using the cation exchange resin (CER) method, as described previously [3,5,11,45,46]. Briefly, 37.5 mL of sludge (equivalent to 1 g suspended solids, SS) was centrifuged at 4000× *g* for 20 min. The pellet was resuspended in 200 mL of deionized water and mixed with 49.7 g of IR120Na cation exchange resin (70 g/g volatile suspended solids, VSS was used to ensure that EPSs were fully extracted) for 4 h under continuous stirring [3,5,11,46]. The mixture was then centrifuged again, and the resulting supernatant was dialyzed (3500 Da molecular weight cutoff) against deionized water for 24 h at a 1:9 (*v*/*v*) ratio. Finally, the dialysate was freeze-dried to obtain EPS powder.

### 2.3. Preparation of BM-M Composite Membranes

**Figure 1:**
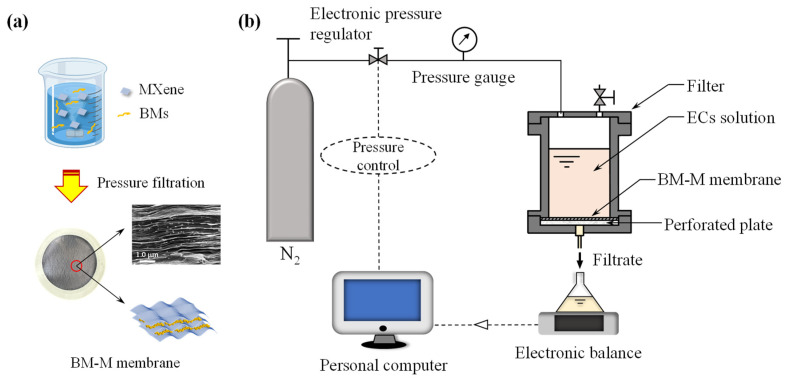
(**a**) Preparation and image of biomacromolecule-intercalated MXene (BM-M) membrane. (**b**) Filtration setup employed for evaluating emerging contaminant (EC) removal and fabricating BM-M membrane by constant-pressure dead-end filtration.

The BM-M composite membranes were fabricated via constant-pressure dead-end filtration with various filtration pressures (defined as the membrane-forming pressure) (Figure 1). First, 50 mg of each BM (EPS, SA, BSA, or SF) was dissolved in 50 mL of deionized water under stirring for 1 h to prepare 1 g·L^−1^ stock solutions. The MXene dispersion (1 g·L^−1^) was then mixed with each BM solution at predetermined mass ratios. The mixtures were sonicated (200 W) for 60 min to ensure homogeneity. The BM-M suspension (60 mL, 1 g·L^−1^) was filtered through a PVDF support membrane (0.22 μm) and air-dried for 6 h to form the BM-M composite membrane (19.6 cm^2^ effective area), denoted as *η*BM-M-*p*. Then, the BM-M composite membrane was carefully peeled off from the PVDF support membrane for subsequent experiments. Here, *η* represents the mass percentage of BM in the composite membrane (0–40%), BM denotes the type of biomacromolecules such as EPS, SA, BSA, and SF, and *p* is the membrane-forming pressure (*p* = 100–500 kPa).

The mass of the permeate was monitored in real-time using an electronic balance connected to a data acquisition system. The filtrate mass was converted to volume based on the density of water, as the low solute concentration allowed this approximation. The filtration rate was determined by numerically differentiating the cumulative filtrate volume with respect to time.

### 2.4. Filtration Experiment of ECs Solution

The removal efficiency of the ECs was assessed by filtering a 10 mg·L^−1^ solution (pH = 6.5) of DEP, ERY, and SMX through the BM-M membranes. This evaluation was carried out at a constant filtration pressure of 100 kPa using the dead-end filtration apparatus depicted in Figure 1b. Each membrane type was tested in triplicate, and the mean values are reported.

### 2.5. Evaluation of Filtration Performance

Parameters such as filtration rate, specific cake resistance, average cake porosity, and EC removal rate were used to evaluate the filtration performance. The dead-end filtration process was analyzed using the Ruth filtration rate equation [47], which posits a linear relationship between the reciprocal of the instantaneous filtration rate (d*θ*/d*v*) and the cumulative filtrate volume per unit area (*v*):

(1) $$\frac{d\theta }{dv}=\frac{2}{K_{v}}+{\left(\frac{d\theta }{dv}\right)}_{m}$$

where *θ* is filtration time, *v* is the cumulative filtrate volume per unit effective membrane area, and (d*θ*/d*v*)_m_ is the reciprocal of the initial filtration rate representing the membrane resistance. *K*_v_ is the Ruth filtration coefficient, defined as:

(2) $$K_{v}=\frac{2p(1-ms)}{\mu \rho s\alpha_{av}}$$

where *p* is the applied filtration pressure (membrane-forming pressure), *m* is the mass ratio of wet cake to dry solids, *μ* is the filtrate viscosity, *ρ* is the filtrate density, and *α*_av_ is the average specific filtration resistance of the cake. *s* is the mass fraction of solids in the suspension (feed concentration), which remains constant in the dead-end pressure process with forming filter cake. For dilute suspensions, (1 − *ms*) ≈ 1, simplifying the equation to:

(3) $$K_{v}=\frac{2p}{\mu \rho s\alpha_{av}}$$

The specific cake resistance (*α*_av_) and average cake porosity (*ε*_av_) were correlated with pressure using empirical power–law relationships (Sperry’s law) [48]:

(4) $$\alpha_{av}=\alpha p^{n}$$

(5) $$1-\varepsilon_{av}=Bp^{\beta }$$

where *α*, *n*, *B*, and *β* are empirical constants, with *n* representing the compressibility coefficient.

The removal rate (*η*) of each EC was calculated from its concentration in the feed (*C*_f_) and percolate (*C*_p_), as determined by a pre-established calibration curve between concentration and UV-Vis absorbance:

(6) $$\eta =\frac{C_{f}-C_{p}}{C_{f}}\times 100\%$$

### 2.6. Analytical Methods

The concentrations of SMX, DEP, and ERY were quantified by UV-Vis spectrophotometry (Cary 5000, Agilent Technologies, Santa Clara, CA, USA) using the calibration curves [10] at their characteristic wavelengths: 267 nm [10], 276 nm [49], and 235 nm [50], respectively. Surface charge (Zeta potential) and colloidal size distribution were analyzed using a nanoparticle analyzer (Delsa Nano C, Beckman Coulter, Brea, CA, USA). Membrane hydrophilicity was assessed via static water contact angle measurements (JC2000D4M, Zhongchen Digital Technology Equipment Co., Ltd., Shanghai, China). They were tested in triplicate, and the mean values were reported. The mechanical properties of the membranes were performed on a universal test machine (AGS-X-50 N, Shimadzu, Kyoto, Japan) with a loading rate of 2.0 mm/min, which was a strip of 8 mm wide and 30 mm long. Young’s modulus was obtained from the slope of the linear region of the stress–strain curve with more than 3 tests. Crystal structures were examined by X-ray diffraction (XRD, Ultima IV, Rigaku Corporation, Tokyo, Japn) with Cu-Kα radiation. Surface functional groups were identified using Fourier transform infrared spectroscopy (FTIR, Nicolet iS5, Thermo Fisher Scientific, Waltham, MA, USA).

## 3. Results and Discussion

### 3.1. Optimization of Membrane-Forming Conditions

**Figure 2:**
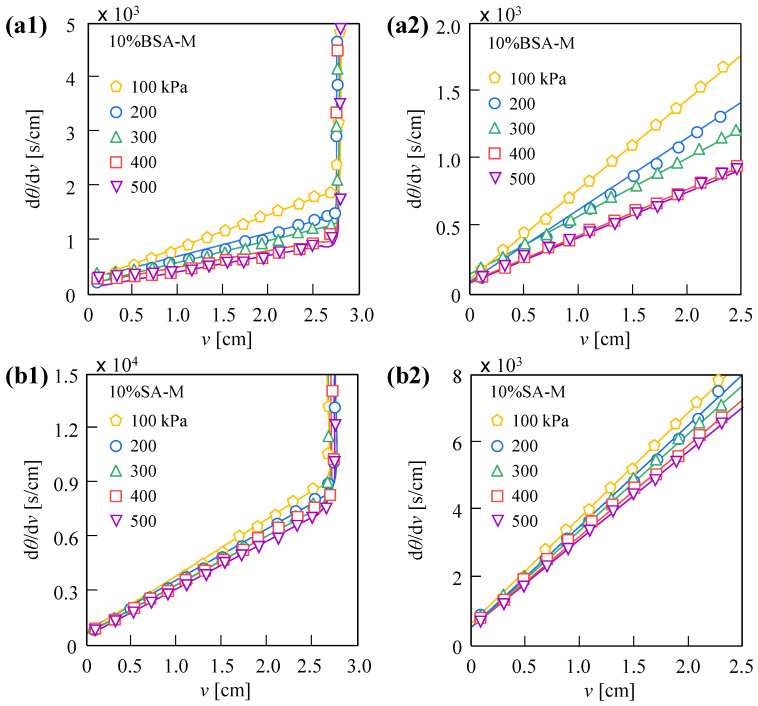
Filtration behaviors of the mixed suspension composed of biological macromolecule and MXene at various membrane-forming pressures (*p*), indicated by the reciprocal filtration rate (d*θ*/d*v*, *θ*: filtration time) versus the filtrate volume per unit effective membrane area (*v*). (**a1**) *v* = 0–3.0 cm and (**a2**) *v* = 0–2.5 cm for the suspension composed of bovine serum albumin (BSA, mass percent of 10%) and MXene nanosheet, 10%BSA-M; (**b1**) *v* = 0–3.0 cm and (**b2**) *v* = 0–2.5 cm for the suspension composed of sodium alginate (SA, mass percent of 10%) and MXene nanosheet, 10%SA-M.

Figure 2(a1,a2) show that for the 10%BSA-M suspension, increasing the membrane-forming pressure (*p* = 100–500 kPa) enhanced the filtration rate (indicated by a decrease in d*θ*/d*v*), with performance plateauing above 400 kPa, likely due to cake compression. In contrast, the 10%SA-M suspension (Figure 2(b1,b2)) exhibited slight improvement in filtration rate with increasing pressure, attributable to the highly compressible “soft structure” of SA. The filtration performance of the 10%SA-M and 10%BSA-M suspensions can be evaluated by Equation (1), due to the linear relationship between d*θ*/d*v* and *v* in the filtration stage, as shown in Figure 2(a2,b2).

**Figure 3:**
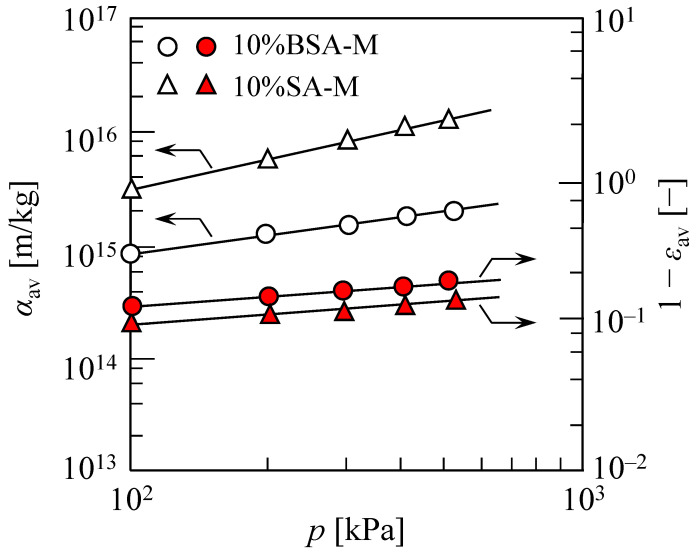
Influence of *p* on the average specific resistance (*α*_av_) and solid volume fraction in filtration cake (1 − *ε*_av_) for 10%BSA-M and 10%SA-M suspensions.

Based on constant-pressure dead-end filtration experiments, the average specific cake resistance (*α*_av_) obtained by Equation (3) and solid volume fraction (1 − *ε*_av_) of 10%BSA-M and 10%SA-M cake are shown in Figure 3. The relationships between *α*_av_ or (1 − *ε*_av_) and *p* can be evaluated by Equations (4) and (5) because of their linear relationship as shown in the figure. Under pressures ranging from 100 to 500 kPa, the solid volume fraction (1 − *ε*_av_) of both suspensions increased with rising pressure, reaching 0.177 for 10%BSA-M and 0.146 for 10%SA-M at 500 kPa, following ${(1-\varepsilon }_{av})=0.062p^{0.16}$ (*R*^2^ = 0.806) and ${(1-\varepsilon }_{av})=0.035p^{0.22}$ (*R*^2^ = 0.897), respectively. However, the *α*_av_ value of 10%BSA-M was lower than that of 10%SA-M, which is attributed to the globular structure of BSA protein, in contrast to the linear chain conformation of SA polysaccharide. Furthermore, the relationship between *α*_av_ and *p* for 10%BSA-M and 10%SA-M can be expressed as: $\alpha_{av}=6.91\times 10^{13}p^{0.55}$ (*R*^2^ = 0.999) and $\alpha_{av}=5.71\times 10^{13}p^{0.88}$ (*R*^2^ = 0.985), respectively, indicating that the former (*n* = 0.55) has a lower compressibility coefficient than the later (*n* = 0.88) [48]. In summary, compared to SA, the use of BSA as a MXene intercalator exhibits reduced filtration resistance during the BM-M composite membrane-forming process and yields a filter cake with enhanced solid content (reduced moisture). Given its superior performance, the 10%BSA-M was selected for further optimization.

**Figure 4:**
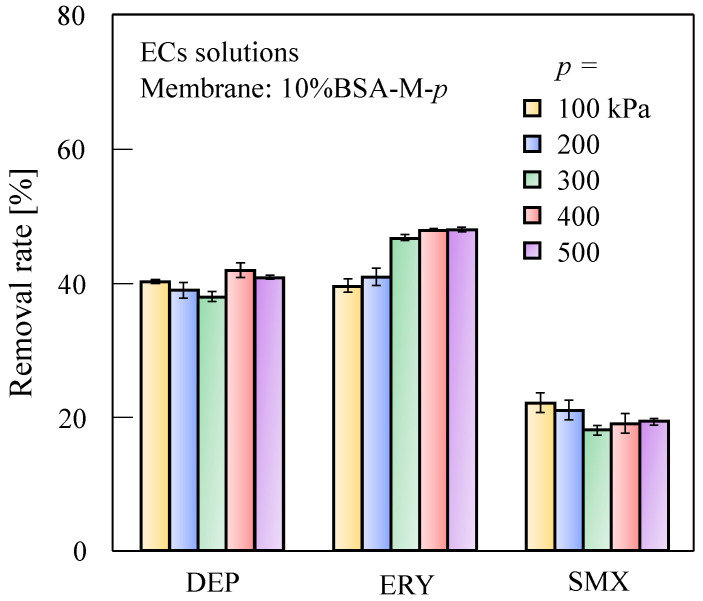
Removal rates of three typical ECs—diethyl phthalate (DEP), erythromycin (ERY), and sulfamethoxazole (SMX)—at filtration with various 10%BSA-M-*p* membranes. Here, 10%BSA-M-*p* denotes the composite membrane formed by 10%BSA-M at the membrane-forming pressure of *p* (=100–500 kPa).

Figure 4 shows the influence of membrane-forming pressure on EC removal using 10%BSA-M-*p* membranes. While the removal of SMX and DEP remained largely unaffected by membrane-forming pressure, a slight enhancement was observed for ERY removal when *p* > 200 kPa. This aligns with the expectation that the largest contaminant molecule (ERY) (Stokes radius of 0.77 nm, as shown in Table 1) would be most sensitive to changes in the compactness and pore structure of the membrane under pressure. Based on the results of Figure 2, Figure 3 and Figure 4, since the effect was modest and lower pressure favors membrane stability and energy efficiency (low filtration resistance), 100 kPa was selected as the optimal membrane-forming pressure for subsequent experiments.

**Figure 5:**
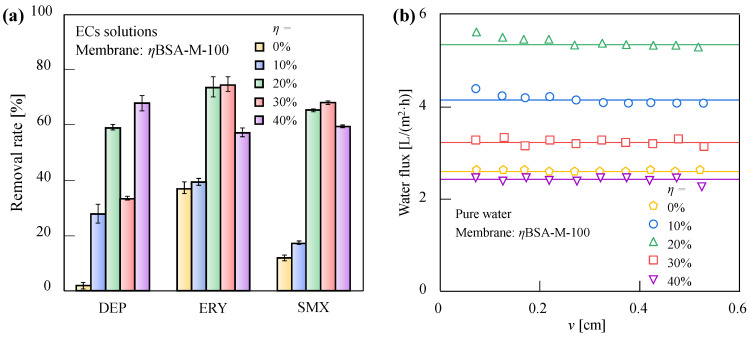
(**a**) Removal rates of three typical ECs and (**b**) pure water flux using *η*BSA-M-100 membranes. Here, *η*BSA-M-100 denotes the composite membrane formed by the suspension composed of BSA (mass percent of *η*) and MXene nanosheet (*η*BSA-M) at the membrane-forming pressure of 100 kPa.

The mass percent of the BM intercalator (*η*) was another critical parameter. Figure 5a demonstrates that the removal efficiency for all three ECs generally improved as the BSA content increased (*η* = 0–40%); however, a further increase in the BSA percentage beyond 20% resulted in only a marginal improvement in removal efficiency. Concurrently, the water flux initially increased significantly when the BSA content rose from 0% to 20% (Figure 5b), likely due to enhanced hydrophilicity and optimized interlayer spacing. However, increasing mass percent of BSA to 40% caused a decline in flux, possibly due to 2D membrane pore blockage. The filtration rate remained constant during experiments, indicating that the 2D membrane remained stable during the filtration process. Based on the balance between high removal efficiency and high water flux, the membrane with BSA of 20% mass percent as intercalation agent was identified as the optimal formulation.

### 3.2. Removal Performance of ECs Using Various BM-M Membranes

**Figure 6:**
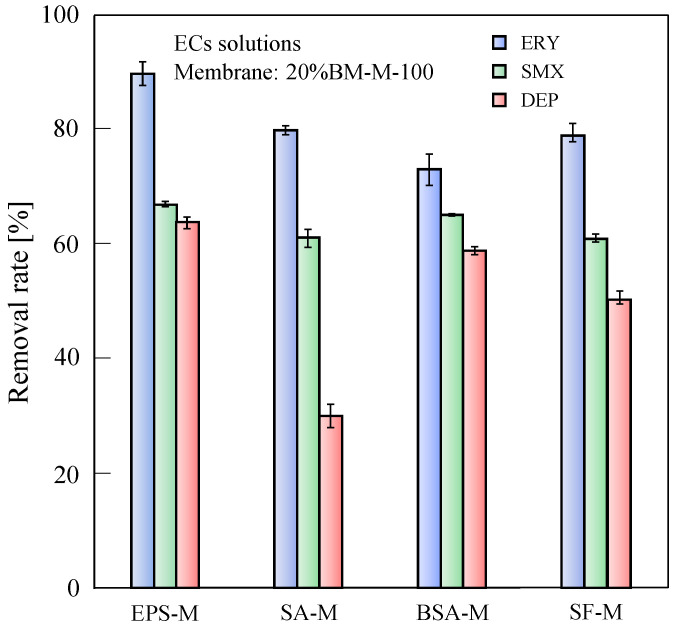
Removal rates of three typical ECs (DEP, ERY, and SMX) using four 20%BM-M-100 membranes. Here, 20%BM-M-100 denotes the composite membrane formed by the suspension composed of BM (mass percent of 20%) and MXene nanosheet (20%BM-M) at the membrane-forming pressure of 100 kPa, in which the BM is practical extracellular polymeric substance (EPS), SA, BSA, or silk fibroin (SF).

The removal capabilities of four BM-M membranes were compared as shown in Figure 6, in which the BM-M membranes were fabricated using the mass fraction of 20% of BM as the intercalator of MXene nanosheet and the membrane-forming pressure of *p* = 100 kPa. The EPS-M membrane exhibited the highest removal efficiencies: 64.0% for DEP, 90.2% for ERY, and 67.5% for SMX, probably because EPS is a mixture composed of multiple biomacromolecules. Overall, removal efficiency correlated positively with the Stokes radius of the contaminants: DEP (smallest) < SMX < ERY (largest) (Table 1) regardless of BM-M membrane types, suggesting a significant size-exclusion mechanism, even though other interactions may also be involved, such as electrostatic and hydrophobic interactions.

**Figure 7:**
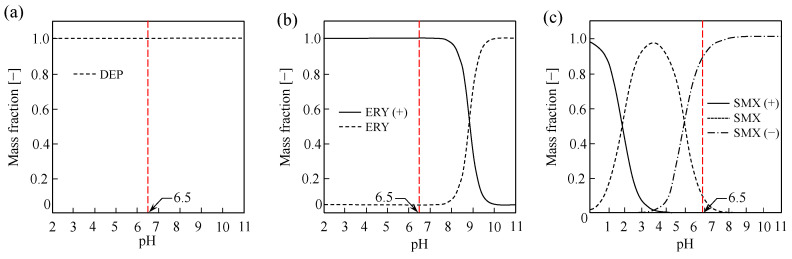
Speciation of cation, molecule and anion as function of pH for (**a**) DEP, (**b**) ERY, and (**c**) SMX [51,52,53]. In this study, the experimental pH = 6.5.

Figure 7 shows the mass fractions of various species of the three ECs as a function of pH. For the smallest and neutral molecule DEP (Table 1 and Figure 7a), the removal rate order was EPS-M > BSA-M > SF-M > SA-M. The low removal by SA-M might be due to DEP’s size being close to its interlayer spacing, allowing penetration. The removal rate order of ERY was EPS-M > SA-M ≈ SF-M > BSA-M (Figure 6). The superior performance of EPS-M can be attributed to its strong negative surface charge, facilitating electrostatic attraction because of the positively charged ERY at pH = 6.5 (Figure 7b), while other BM-M membranes’ performance has little differences. For the mainly negatively charged SMX (Figure 7c), BM-M membrane types has litter effect for the removal rate of SMX despite their structural differences, suggesting that electrostatic repulsion between the negatively charged membranes and SMX played a significant role, potentially overshadowing membrane structural effects. 

### 3.3. Characteristics of Different BM-M Membranes

**Figure 8:**
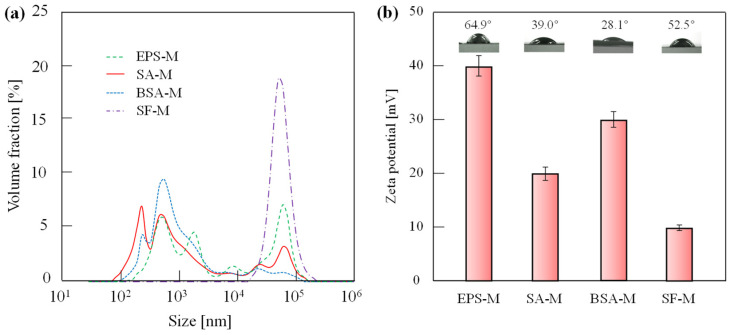
(**a**) Size distribution of suspended colloids in the suspension composed of various BMs (mass percent of 20%) and MXene nanosheet (20%BM-M). (**b**) Zeta potential and water contact angle of four corresponding BM-M membranes.

Figure 8a shows the size distributions of suspended colloids in the suspensions composed of various BMs (mass percent of 20%) and MXene nanosheet. The average sizes of EPS-M, SA-M, BSA-M, and SF-M are 2.08, 0.70, 0.70, and 51.57 μm, respectively. The pure water flux of four membranes followed the order: SF-M [2.5 L/(m^2^·h)] < SA-M [2.8 L/(m^2^·h)] < EPS-M [2.9 L/(m^2^·h)] < BSA-M [5.4 L/(m^2^·h)]. However, this result cannot be explained by the average size of the colloids. Generally, the larger the size, the greater the membrane porosity, and the greater the membrane water flux. Therefore, this might also depend on the shape of the BM intercalators and the charge properties of their interface. BSA-M’s highest flux is consistent with its spherical structure creating a more open and porous membrane matrix. However, EPS-M’s superior contaminant removal highlights that its performance stems from an optimal combination of surface properties and structure. The presence of spherical proteins between the MXene nanosheets may be beneficial for the formed BM-M membrane’s removal of small molecular pollutants (Figure 6 for DEP).

The Zeta potential and water contact angle (WCA) of four corresponding BM-M membranes are shown in Figure 8b, with error bars representing the standard deviation from three replicates. EPS-M exhibited the strongest negative surface charge (−40.2 mV), significantly enhancing its adsorption capacity for positively charged contaminants (e.g., ERY). Additionally, EPS-M demonstrated stronger hydrophobicity, further improving its removal efficiency for hydrophobic positively charged pollutants such as ERY. Although BSA-M also carried a strong negative charge (−30.2 mV), its high hydrophilicity resulted in weaker performance for hydrophobic positively charged contaminants (e.g., ERY). SA-M had weaker negative charge (−20.3 mV) and moderate hydrophilicity (WCA of 39°), leading to limited removal capability for ERY. SF-M, with its relatively weak surface charge (−10.1 mV), exhibited limited adsorption capacity for positively charged contaminants and may be more suitable for removing neutral or hydrophobic pollutants.

In addition, as shown in Figure 8b, the BSA-M membrane exhibited the smallest WCA, indicating stronger surface hydrophilicity. This weakened the interaction between the membrane and water-soluble contaminants (e.g., ERY), thereby reducing their removal efficiency (Figure 6). The strong hydrophilicity also reduced hydraulic resistance, contributing to the highest water flux [5.4 L/(m^2^·h)]. In contrast, the SF-M membrane showed lower water flux [2.5 L/(m^2^·h)], likely due to its weak surface charge (Figure 8b) and high membrane resistance. Notably, the EPS-M membrane with the highest WCA (indicating stronger hydrophobicity), may have impeded water permeation, further increasing the removal rate of ECs (Figure 6).

**Figure 9:**
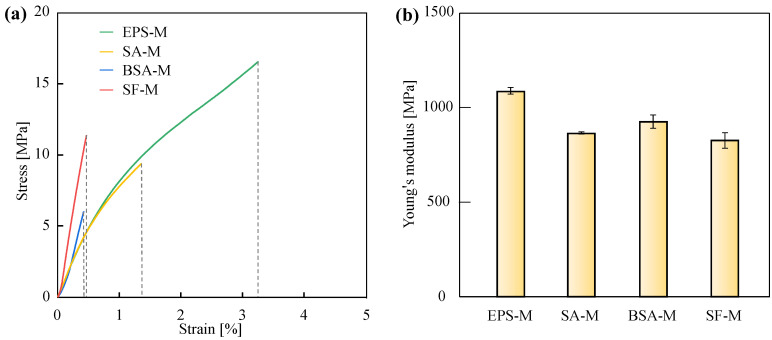
(**a**) Typical stress–strain curves and (**b**) the corresponding Young’s modulus of the four BM-M membranes.

Figure 9 shows the typical stress–strain curves and the corresponding Young’s modulus of the four BM-M membranes. The results revealed significant differences in ultimate tensile strength and Young’s modulus among the four composite membranes. EPS-M exhibited the superior mechanical performance, with an ultimate tensile strength of 15.2 MPa and a Young’s modulus of 1088.7 MPa. This exceptional performance can be attributed to the dense and heterogeneous structure of practical EPS, which contains polysaccharides, proteins, and nucleic acids, endowing it with high resistance to deformation. SA-M exhibited better ductility than BSA-M, demonstrating moderate flexibility. SF-M demonstrated high strength (11.40 MPa) but poor ductility, indicating brittle behavior. BSA-M showed the lowest strength (6.02 MPa) among the membranes, with a relatively fragile structure, though its rigidity was slightly higher than that of SA-M. In summary, EPS-M outperformed other membranes in strength, rigidity, and ductility, making it a promising candidate for practical applications.

**Figure 10:**
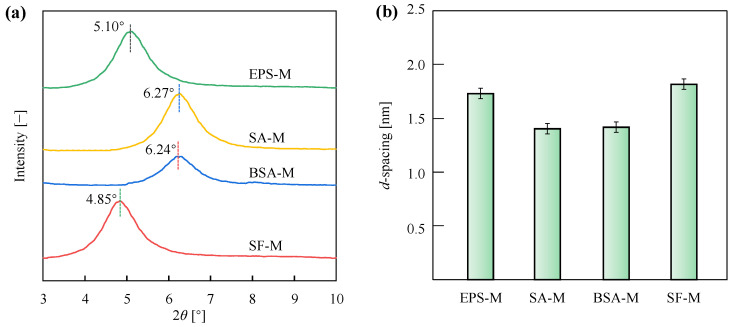
(**a**) X-ray diffraction patterns and (**b**) the corresponding *d*-spacing of the four BM-M membranes.

Figure 10 shows XRD patterns and *d*-spacing of the four BM-M membranes. The MXene diffraction peak at 6.55° [3] showed significant shifts when intercalated with EPS or SF, decreasing to 5.10° and 4.85°, respectively, while SA-M and BSA-M membranes exhibited no notable peak changes. This indicates that EPS and SF can expand the interlayer spacing of MXene through physical or chemical interactions such as hydrogen bonding, ion exchange, or electrostatic forces. The interlayer distances (*d*-spacing) between MXene nanosheets in EPS-M membranes were calculated using the Bragg’s law [3]: 2*d*sin*θ* = *nλ*, where *λ* is the wavelength of the incident wave (=0.154 nm) (*n* = 1), *θ* is the angle between the incident wave and the scattering; *d* is the apparent interlayer spacing. As shown in Figure 10b, the *d*-spacing values of EPS-M, SA-M, BSA-M, and SF-M were 1.73, 1.41, 1.42, and 1.82 nm, respectively. The larger interlayer spacing for EPS-M and SF-M membranes suggests that EPS and SF can form stronger interactions with MXene surfaces through their molecular structures, intercalating into MXene layers and increasing the interlayer spacing.

**Figure 11:**
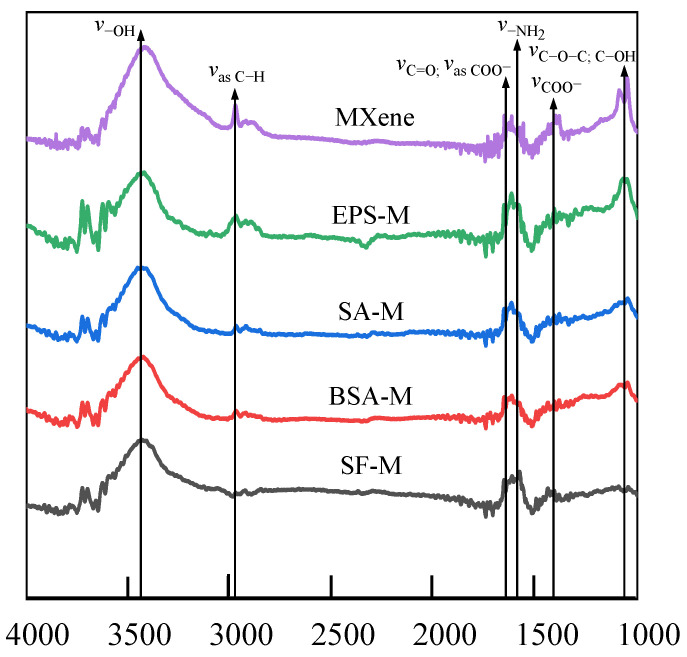
Fourier transform infrared spectrometer (FTIR) of the pristine MXene and four BM-M membranes.

Figure 11 shows the FTIR spectra of pristine MXene [3] and four BM-M composite 2D membranes, which identified characteristic absorption peaks at 3400 cm^−1^ (*v*_–OH_), 2970 cm^−1^ (*v*_as C–H_), 1606 cm^−1^ (*v*_C=O_; *v*_as-COO_^−^), 1575 cm^−1^ (*v*_−NH_2__), 1400 cm^−1^ (*v*_–COO_^−^), and 1055 cm^−1^ (*v*_C–O–C; C–OH_) [54,55]. Compared to pristine MXene membrane [3], all four composite membranes exhibited significant shifts in the –OH peaks, indicating hydrogen bonding interactions between the BMs and MXene surfaces. Functional group analysis revealed that –OH and −NH_2_ groups could interact with polar pollutants (e.g., SMX) through hydrogen bonding, while carboxylate groups (–COO^−^) potentially adsorbed positively charged pollutants (e.g., ERY). Hydrophobic groups (–C–H) contributed to hydrophobic interactions with nonpolar pollutants (e.g., DEP), enabling simultaneous removal of diverse contaminant types. The ATR-FTIR spectra demonstrated distinct features among the four membranes: BSA-M and SF-M, both protein-based membranes, exhibited similar functional groups but differed in peak intensities due to structural variations. SA-M, a polysaccharide-rich membrane, lacked −NH_2_ groups but contained abundant –OH and –COO^−^ groups, favoring electrostatic adsorption of ERY. BSA-M showed strong −NH_2_ and –COO^−^ peaks, enhancing adsorption of polar pollutants such as SMX. EPS-M, with its complex composition including protein, polysaccharide, humic substances and nucleic acids, displayed prominent –COO^−^ and –C–H groups, enabling simultaneous removal of charged and hydrophobic pollutants. SF-M relied primarily on hydrogen bonding for SMX adsorption but exhibited weaker performance for hydrophobic DEP due to limited –C–H groups. These functional group characteristics determine the selective removal mechanisms of the composite membranes, providing theoretical guidance for optimizing membrane design.

Furthermore, according to the results of *d*-spacing (Figure 10b) and FTIR (Figure 11), the large interlayer spacing in EPS-M may be attributed to intercalation via hydrogen bonding and electrostatic attraction between EPS and MXene layers. As a protein material, SF’s amino and carboxyl groups may interact with MXene surfaces, forming larger interlayer spacing. The protein chain structure could further increase the interlayer spacing, thereby enhancing the membrane’s adsorption capacity for certain contaminants (e.g., charged contaminants). In contrast, SA-M and BSA-M exhibited smaller interlayer spacing due to fewer charged groups and weaker interactions, resulting in less noticeable interlayer changes. The larger interlayer spacing in EPS-M and SF-M suggests superior removal capabilities for larger or charged pollutants, which is consistent with their high removal rates for SMX and ERY among the three ECs tested. Detailed analytical data including XRD, FTIR, SEM, and XPS for the pristine MXene membrane and EPS-M membrane can be found in our previously published literature [3].

### 3.4. Analysis of Emerging Contaminant Removal Mechanism

**Figure 12:**
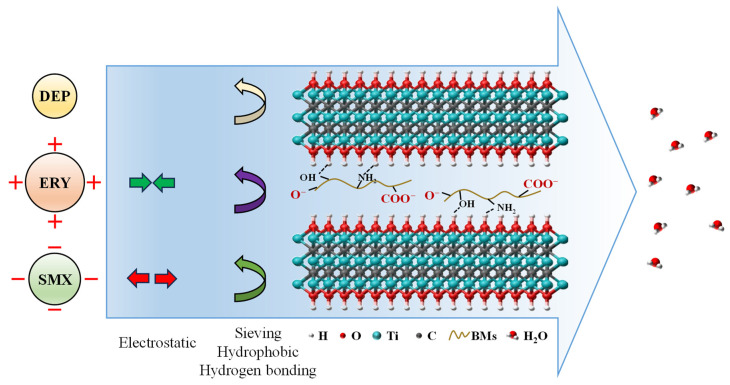
Removal mechanisms of the three ECs (DEP, ERY, and SMX) during filtration with BM-M membranes.

The enhanced removal of ECs by BM-M membranes is achieved through pollutant-specific pathways, which are programmed by the functional groups and structural features of the BM intercalators. The dominant mechanism for each contaminant arises from a synergistic combination of size exclusion, electrostatic interactions, hydrogen bonding, and hydrophobic effects (Figure 12), as empirically dictated by the membrane’s tuned properties (Figure 8, Figure 9, Figure 10 and Figure 11).

For ERY, the dominant mechanism is electrostatic attraction synergized with hydrophobic interaction. The superior performance of the EPS-M membrane (90.2% removal) is directly attributed to its strongest negative surface charge (Zeta potential: −40.2 mV, Figure 8b) and moderate hydrophobicity, originating from its abundant –COO^−^ and –C–H groups (FTIR, Figure 11). For SMX, electrostatic repulsion from the negatively charged membrane surfaces (Figure 7c) is the primary rejection force, explaining the comparable removal efficiencies across most membranes (Figure 6). This is complemented by hydrogen bonding with –OH/−NH_2_ groups present in intercalators like BSA and EPS (Figure 11). For DEP, removal relies on hydrophobic interactions and size exclusion. The highest DEP removal by EPS-M (64.0%) correlates with its relatively hydrophobic character (Figure 8b) and prominent –C–H groups (Figure 11), which facilitate the adsorption of this neutral, hydrophobic contaminant. In summary, the strategic selection of a BM intercalator tailors the membrane’s interfacial properties (charge, hydrophobicity, functional groups), thereby activating a specific combination of mechanisms for the targeted and enhanced removal of each pollutant.

## 4. Conclusions

This work establishes a sustainable modification strategy of 2D MXene membrane by valorizing EPS recovered from excess sludge. Various BM-M membranes using practical and model EPSs as intercalation agents of MXene nanosheets were fabricated and used to separate three typical ECs such as DEP, ERY, and SMX. Considering membrane stability, energy efficiency, removal rate of ECs, and water flux, the membrane-forming pressure of 100 kPa and BMs’ mass precent of 20% were suggested. Practical EPS enabled the most balanced membrane performance and contaminant removal followed a clear size-dependent trend, confirming size exclusion as the dominant separation mechanism. The EPS-M membrane achieved removal of structurally diverse contaminants, with representative removal efficiencies exceeding 60% for small neutral molecules and approaching 90% for large, positively charged compounds, while maintaining stable water permeability. This enhanced performance is associated with the combined effects of enlarged interlayer spacing, a strongly negative surface charge, and improved mechanical integrity. The practical EPS interlayer agent performed better than the model EPS, highlighting the importance of molecular complexity in interlayer design. Future studies should evaluate mechanistic interpretation, microscopic structure and interfacial characterization of membrane, liquid environment, long-term stability and fouling resistance under realistic conditions (e.g., trace concentration, multicomponent ECs), and optimize EPS composition to enhance membrane selectivity.

## Figures and Tables

**Table 1:** Molecular structure and physicochemical properties of three typical ECs such as diethyl phthalate (DEP), erythromycin (ERY), and sulfamethoxazole (SMX).

Compound	MW [g/mol]	Molecular Formula	p*K*_a_ *		Log*K*_ow_ *	Stokes Radius [nm]
			p*K*_a1_	p*K*_a2_		
DEP	222.24	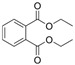	8	-	2.47	0.27 [43]
ERY	733.94	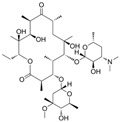	8.8	-	3.06	0.77 [44]
SMX	253.28	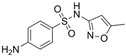	1.8	5.6–5.7	0.89	0.39 [12]

* The values are sourced from: https://pubchem.ncbi.nlm.nih.gov/.

## Data Availability

The original contributions presented in this study are included in the article. Further inquiries can be directed to the corresponding author.

## Nomenclature

*B*	empirical constant defined in Equation (5)	[kg^−*β*^·m*^β^*·s^2*β*^]
*C_f_*	concentration of emerging contaminant in feed solution	[g/L]
*C_p_*	concentration of emerging contaminant in percolate	[g/L]
*K_v_*	Ruth filtration coefficient defined by Equation (1)	[m^2^/s]
*m*	mass ratio of wet cake to dry solids	[−]
*n*	compressibility coefficient	[−]
*p*	filtration pressure	[kPa]
*s*	mass fraction of solids in suspension	[−]
*v*	cumulative filtrate volume per unit effective membrane area	[m]
*Greek letters*		
*α*	empirical constant defined in Equation (4)	[kg^−1−*n*^·m^1+*n*^·s^2*n*^]
*α_av_*	average specific cake resistance	[m/kg]
*β*	empirical constant defined in Equation (5)	[−]
*η*	removal efficiency of emerging contaminant	[%]
*ε_av_*	average cake porosity	[−]
*θ*	filtration time	[s]
*μ*	filtrate viscosity	[Pa·s]
*ρ*	filtrate density	[kg/m^3^]
Subscripts		
*m*	membrane	
